# Suggesting *Dictyostelium* as a Model for Disease-Related Protein Studies through Myosin II Polymerization Pathway

**DOI:** 10.3390/cells13030263

**Published:** 2024-01-31

**Authors:** Xiong Liu, Shi Shu

**Affiliations:** Laboratory of Cell Biology, National Heart, Lung, and Blood Institute (NHLBI), National Institutes of Health (NIH), Bethesda, MD 20892, USA; shis@nhlbi.nih.gov

**Keywords:** *Dictyostelium*, myosin II, polymerization, electron microscopy

## Abstract

*Dictyostelium* myosin II displays remarkable dynamism within the cell, continually undergoing polymerization and depolymerization processes. Under low-ion conditions, it assumes a folded structure like muscle myosins and forms thick filaments through polymerization. In our study, we presented intermediate structures observed during the early stages of polymerization of purified myosin via negative staining electron microscopy, immediately crosslinked with glutaraldehyde at the onset of polymerization. We identified folded monomers, dimers, and tetramers in the process. Our findings suggest that *Dictyostelium* myosin II follows a polymerization pathway in vitro akin to muscle myosin, with folded monomers forming folded parallel and antiparallel dimers that subsequently associate to create folded tetramers. These folded tetramers eventually unfold and associate with other tetramers to produce long filaments. Furthermore, our research revealed that ATP influences filament size, reducing it regardless of the status of RLC phosphorylation while significantly increasing the critical polymerization concentrations from 0.2 to 9 nM. In addition, we demonstrate the morphology of fully matured *Dictyostelium* myosin II filaments.

## 1. Introduction

The myosin superfamily comprises 35 diverse classes of proteins, with Class 2, also known as conventional myosins, being extensively studied [1,2,3]. This class includes muscle myosin, mammalian nonmuscle myosin IIs, and myosin IIs from various organisms like *Dictyostelium*, *Acanthamoeba*, yeast, and fungi [3,4]. A common structural feature among myosin IIs is their hexameric monomer structure, consisting of two long heavy chains (HCs), two short regulatory light chains (RLCs), and two short essential light chains (ELCs) [5]. The HCs include a globular motor domain (head) with actin-activated ATPase activity, a lever arm (neck) that binds an RLC and an ELC, and a long helical tail that homodimerizes into a coiled coil. All myosin IIs also possess an assembly domain.

Nonmuscle myosin IIs are highly dynamic within the cell, disassembling and reassembling at different locations to perform various functions like cell division, adhesion, migration, endocytosis, exocytosis, and tissue morphogenesis [4,6]. Understanding the polymerization and depolymerization mechanisms of myosin IIs is crucial for comprehending their physiological roles.

Recent studies have revealed species-specific variations in the polymerization pathways of myosin IIs, despite their common structural features. Huxley’s model, proposed in the 1950s for muscle myosin II polymerization, involved the formation of antiparallel dimers through the assembly domain in the heavy chain’s C-terminus. These dimers are then associated to create bipolar filaments with head staggering [7,8]. This mechanism was observed in *Acanthamoeba* myosin II [9]. However, our recent research showed that muscle myosin IIs and mammalian nonmuscle myosin IIs follow a distinct assembly pathway [10,11]. They first form folded monomers with three segments of the long tail that associate into antiparallel folded dimers, further creating antiparallel folded tetramers without head staggering. These folded tetramers then associate, unfold, and form bipolar filaments.

*Dictyostelium* myosin II was initially purified by Clark M and Spudich JA in 1974 [12], marking the beginning of extensive research on this protein. Since the late 1980s, it has been a model for exploring the relationship between myosin II structure and function due to its genetic manipulability [13]. The heavy chain of *Dictyostelium* myosin II, comprising 2116 amino acid residues, forms dimers with a tail length of 186 nm [14,15]. Under low-ionic-strength conditions, it spontaneously assembles into thick filaments, being soluble in zero salt and 200 mM KCl buffer, with maximum polymerization observed around 50 mM KCl [16,17]. Rotary shadowing EM studies revealed various structures, including extended monomers, antiparallel dimers, parallel dimers, antiparallel tetramers, and parallel tetramers [15,16,17], in addition to filaments. The parallel dimers were also observed under negative staining EM [18]. The polymerization pathway is proposed based on parallel dimer structures in the polymerization solution: monomers form parallel dimers that associate into extended antiparallel tetramers, and filaments grow by adding parallel dimers via lateral association [19,20].

The tail of muscle myosin and mammalian nonmuscle myosin IIs fold into three segments that bend toward the heads in low-salt conditions, interacting with the heads. At the same time, the distal section flanks them [21]. A recent study reveals that *Dictyostelium* myosin II monomers adopt a folded structure in low-salt conditions, similar but not identical to their mammalian counterparts [22]. While the initial bend of the *Dictyostelium* myosin II tail is like that of animal myosin IIs, the tail extends beyond the heads, folding one or more times on the opposite side of the heads. This observation challenges the previously proposed polymerization pathway for *Dictyostelium* myosin II [19,20].

In our study, we investigate how *Dictyostelium* myosin II polymerizes in vitro and analyze the effects of light chain phosphorylation and the presence of ATP on its assembly. Our data indicate that *Dictyostelium* myosin II shares the polymerization pathway with muscle myosin. This suggests that some *Dictyostelium* proteins may closely resemble their counterparts in human cells, implying the conservation of evolved features in human cells.

## 2. Material and Methods

### 2.1. Purification of Dictyostelium Myosin II 

The RLC phosphorylated and non-RLC phosphorylated *Dictyostelium* myosin II were prepared in a previous study and have since been stored in liquid nitrogen, with RLC being fully phosphorylated, as confirmed by urea-glycerol polyacrylamide gel electrophoresis [23]. It was dissolved in 250 mM NaCl and 10 mM MOPS (pH 7.0) and stored in the liquid N2 until use. 

### 2.2. Protein Concentration Assay

Protein concentrations were determined using the Bradford reagent (Bio-Rad, Hercules, CA, USA) with purified myosin as standard. The concentration of the myosin standard was determined by UV absorbance and calculated according to the formula: [myosin] (mg/mL) = (*A*_280_ – *A*_320_)/0.52. 

### 2.3. Light Scattering Assay of Myosin Assembly

Myosin samples in 250 mM NaCl were cleared by centrifugation at 300,000× *g* for 10 min at 4 °C in a Beckman TL-100 centrifuge (Beckman Coulter, Indianapolis, IN, USA) before use. Myosins were polymerized overnight on ice after dilution with appropriate buffers, as described in the figure legends. The samples were warmed to room temperature for 30 min and light scattering was measured at 20 °C in a PTI fluorimeter (Horiba Scientific, Kyoto, Japan). Excitation was performed at 365 nm (slit width 0.5 nm) and detected at 365 nm (slit width 0.5 nm). 

### 2.4. Electron Microscopy

Protein samples were cleared before use by centrifugation at 300,000× *g* for 10 min at 4 °C in a Beckman TL-100 centrifuge (Beckman Coulter, Indianapolis, IN, USA). Myosin (except for RLC-unphosphorylated myosins + ATP) were polymerized in 10 mM MOPS, pH 7.0, 50 mM NaCl, 2 mM MgCl_2_, with or without 1 mM ATP. Polymerization was initiated by dilution and 0.1% glutaraldehyde was immediately added to fix the structures for 5 min, and 5 µL of the sample was applied to a UV-light-pretreated carbon-coated copper grid and stained with 1% uranyl acetate. Micrographs were recorded on a JEOL 1200EX II microscope (Peabody, MA, USA) at room temperature. Filament widths and lengths were determined with Metamorph software (version I).

## 3. Result

### 3.1. Morphology of Mature Dictyostelium Myosin II Filaments with and without ATP

**Figure 1:**
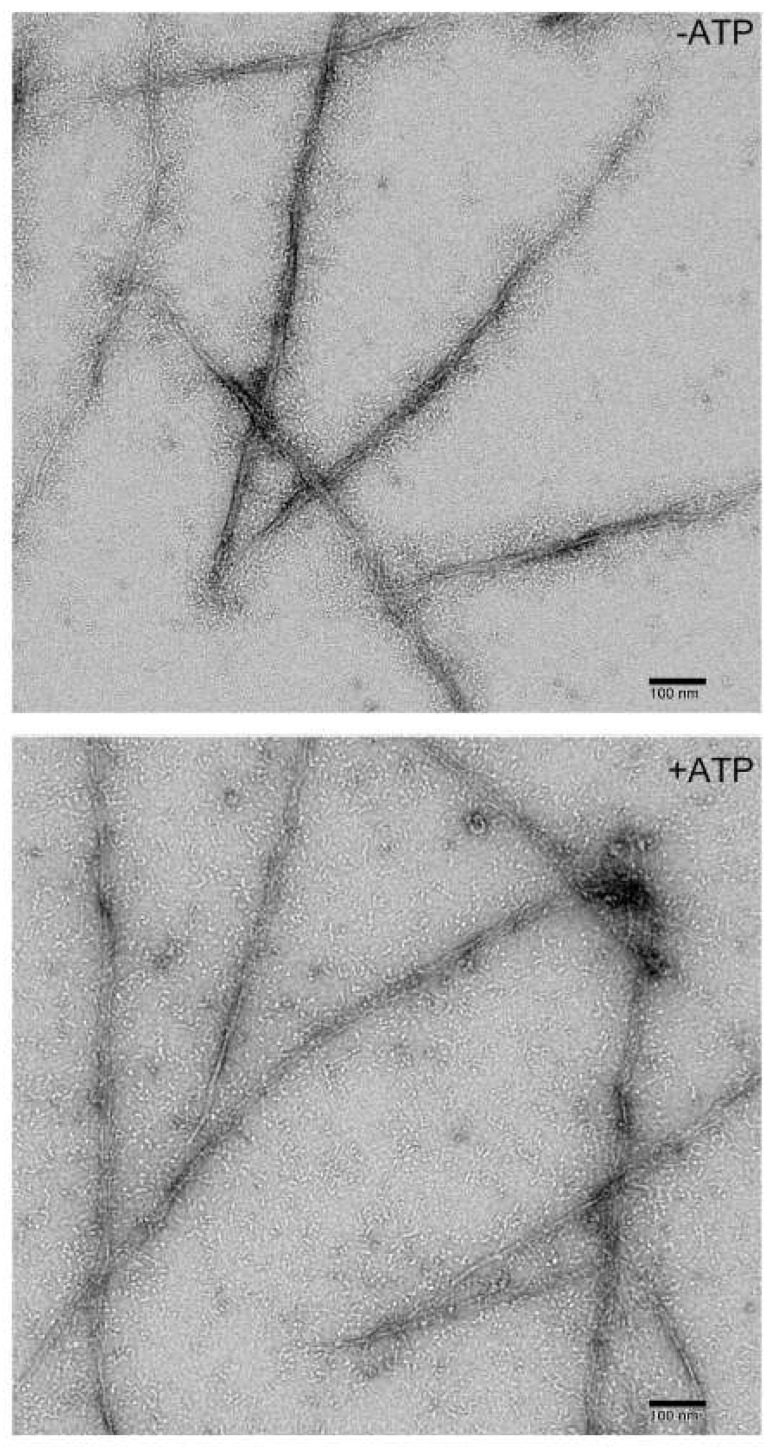
Field images of overnight polymerized *Dictyostelium* myosin II with and without 1 mM ATP. *Dictyostelium* myosin II was polymerized in the presence of 10 mM MOPS, 2 mM MgCl_2_, and 50 mM NaCl.

**Figure 2:**
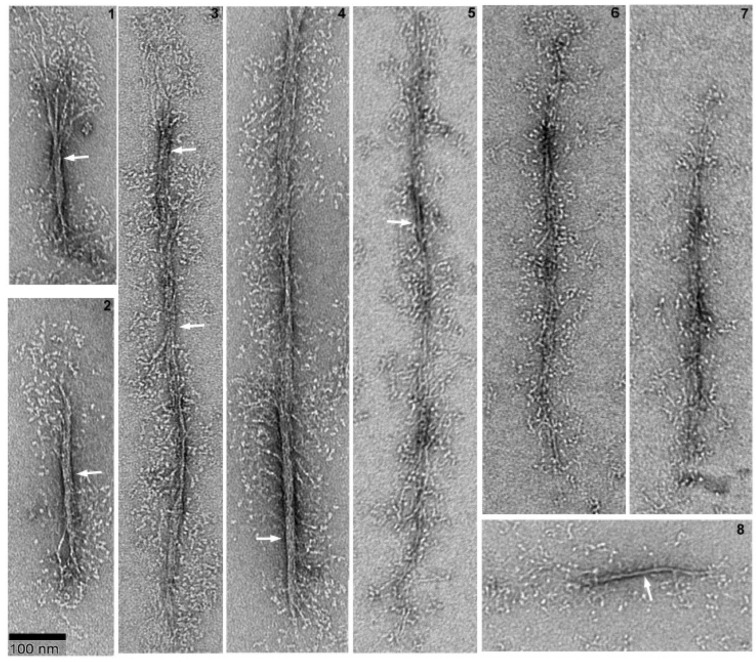
Selected filaments of overnight polymerized *Dictyostelium* myosin II with and without 1 mM ATP. Myosin was polymerized in the presence of 10 mM MOPS, 2 mM MgCl_2_, and 50 mM NaCl. Panels 1 to 4 show myosin filaments polymerized without ATP and panels 5 to 8 indicate filaments polymerized with ATP. White arrows indicate bare zones.

To achieve complete polymerization, myosin was polymerized overnight, both in the presence and absence of ATP. Field images are presented in Figure 1, while Figure 2 showcases selected filament images. Without ATP, myosin forms long, slender filaments of varying widths with extended heads. The filament sizes (width and length) display significant heterogeneity, and some filaments exhibit bare zones, including ATP in the polymerization buffer, resulting in thinner and smaller filaments with sparser head distribution. This suggests that ATP inhibits *Dictyostelium* myosin II polymerization. Some bare zones are evident in the mature filaments, as indicated by white arrows. Our images depict the clear filament structure and head distribution, which is like its mammalian counterpart. The formation of these long filaments seems to result from tandem connections of short filaments. 

Filament dimensions with ATP measure 495.6 nm ± 130.3 (n = 32) in length and 4.9 nm ± 2.3 (n = 50) in width. In the absence of ATP, filament length and width are 561.8 ± 148.8 nm (n = 60) and 10.5 ± 2.6 nm (n = 55), respectively, with lengths ranging from 370 to 1100 nm. These data demonstrate a greater impact of ATP on filament width. The polymerized filament lengths are consistent with measurements from total internal reflection fluorescence (TIRF) microscopy (680 nm, 400 to 950 nm) and thin-section TEM (500 nm, 400 to 600 nm) in our experimental conditions [24,25]. Filaments longer than 600 nm in thin-section TEM observation [20] could be missed due to disassembly during fixation [26]. Figure 1 and Figure 2 showcase long filaments formed in our conditions, yet shorter bipolar filaments, as depicted in panels 1, 2, and 8 of Figure 2, are also prevalent, typically exhibiting a bipolar configuration with a bare zone.

### 3.2. Determination of Critical Polymerization Concentration

**Figure 3:**
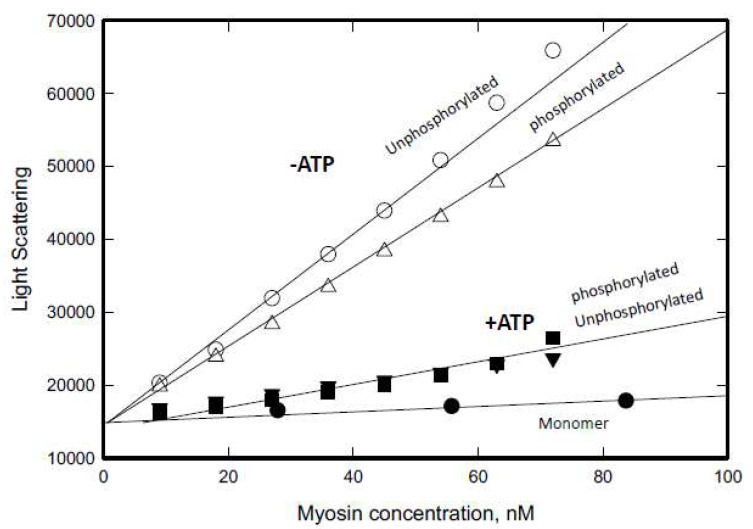
The minimal concentrations required for polymerization (critical concentration) of non-RLC and RLC-phosphorylated *Dictyostelium* myosin II with and without 1 mM ATP determined by light scattering. Myosin was incubated overnight on ice in 50 mM NaCl, 10 mM Mops (pH 7.0), and 2 mM MgCl_2_, and with or without 1 mM ATP. Myosin monomer was prepared by centrifugation of myosin in buffer containing 250 mM NaCl at 300,000× *g* for 15 min. The critical concentrations are the intercepts of the light scattering plots of the assembled myosin with the light scattering plot of non-RLC phosphorylated monomer. Light scattering data were plotted by Sigma Plot (V15). Open circle: non-RLC phosphorylated in the absence of ATP; open triangle: RLC phosphorylated in the absence of ATP; closed square: non-RLC phosphorylated in the presence of ATP; closed triangle: RLC phosphorylated in the presence of ATP; closed circle: the myosin monomer (non-RLC phosphorylated in 250 mM NaCl). Data are the averages of two assays.

To quantitatively assess the impact of ATP on polymerization, we measured the light scattering intensity of myosin with and without RLC phosphorylation. As reported previously [19], Dictyostelium myosin II polymerization is rapid, with half-times for maximum polymerization at concentrations of 100 nM, 150 nM, 250 nM, and 300 nM being 12 min, 5 min, 1 min, and 0.5 min, respectively. Samples in this assay were polymerized overnight at 4 °C to ensure full polymerization. Notably, myosin displayed similar light scattering patterns irrespective of RLC phosphorylation status in both ATP conditions. However, the presence of ATP led to a significant reduction in light scattering, indicating a notable decrease in the size of myosin filaments, consistent with the morphology shown in Figure 2 and Figure 3.

Previously, Kaczmarski et al. [18] determined the critical polymerization concentration using airfuge centrifugation to be approximately 18 nM without ATP. Our findings show that the critical polymerization concentrations are approximately 0.2 nM in the absence of ATP and 9 nM in the presence of ATP for both RLC-phosphorylated and RLC non-phosphorylated forms. This represents a 45-fold increase in the critical concentration in the presence of ATP. The significant difference from Kaczmarski et al.’s data likely arises from the greater sensitivity of the light scattering method compared to centrifugation assays. Notably, our data also indicate that RLC phosphorylation does not affect the critical concentration.

We had previously determined the critical polymerization concentrations for muscle and mammalian nonmuscle myosin IIs [11,27]. Skeletal muscle myosin and cardiac muscle myosin were minimally affected by ATP in the polymerization buffer, with critical concentrations of 6 and 13 nM in the presence of ATP and 4 and 7 nM in the absence of ATP (Table 1). In contrast, smooth muscle myosin 2 was substantially affected by ATP, with critical concentrations of 117 nM in the presence of ATP and 6 nM in the absence of ATP (Table 1). For mammalian nonmuscle myosin 2A, 2B, and 2C, the critical concentrations were 10 nM, 7 nM, and 3 nM in the absence of ATP and 80 nM, 50 nM, and 70 nM, respectively, in the presence of ATP (Table 1). These findings highlight that *Dictyostelium* myosin II exhibits the lowest critical concentration in the absence of ATP. In the presence of ATP, its critical concentration is like that of skeletal and cardiac muscle myosin. Among all of these myosin II variants, ATP has the most significant effect on the critical concentration of *Dictyostelium* myosin II.

### 3.3. Intermediate Structures during Polymerization

**Figure 4:**
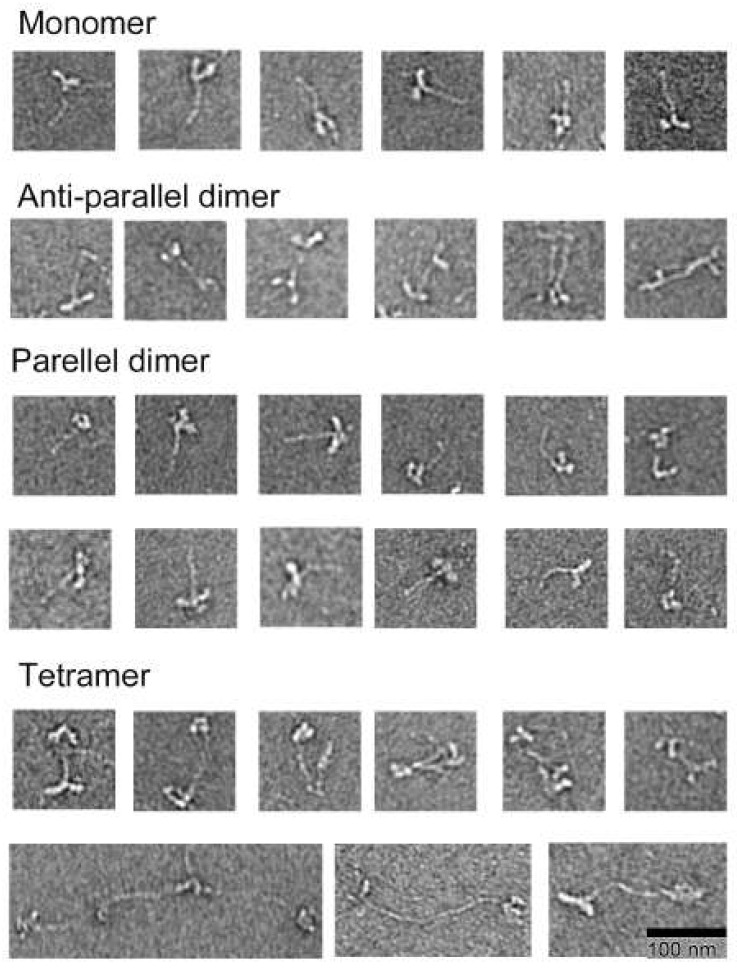
Electron micrographs of the intermediate structures formed during polymerization. *Dictyostelium* myosin II formed folded monomers, folded antiparallel dimers, folded parallel dimers, and folded antiparallel tetramers. The folded tetramers had varied lengths.

For myosin polymerization, direct dilution was employed to reach conditions of 2 mM MgCl_2_ and 50 mM NaCl, followed by immediate fixation with 0.1% glutaraldehyde and application to grids. In this context, four distinct intermediate structures were observed (Figure 4). These structures encompass folded monomers, folded parallel and antiparallel dimers, folded antiparallel tetramers, and partially folded antiparallel tetramers. Of note, at the onset of polymerization, the majority of small structural species are folded monomers. These folded monomers, along with dimers and tetramers, constitute 96%, 3.5%, and 0.5%, respectively.

Notably, except for folded monomers, these structures have yet to be reported previously in *Dictyostelium*. The presence of partially folded antiparallel tetramers suggests that folded structures undergo unfolding at the tetramer structural level. 

### 3.4. Association of Folded Tetramers with Growing Filaments and Polymerization Pathway

**Figure 5:**
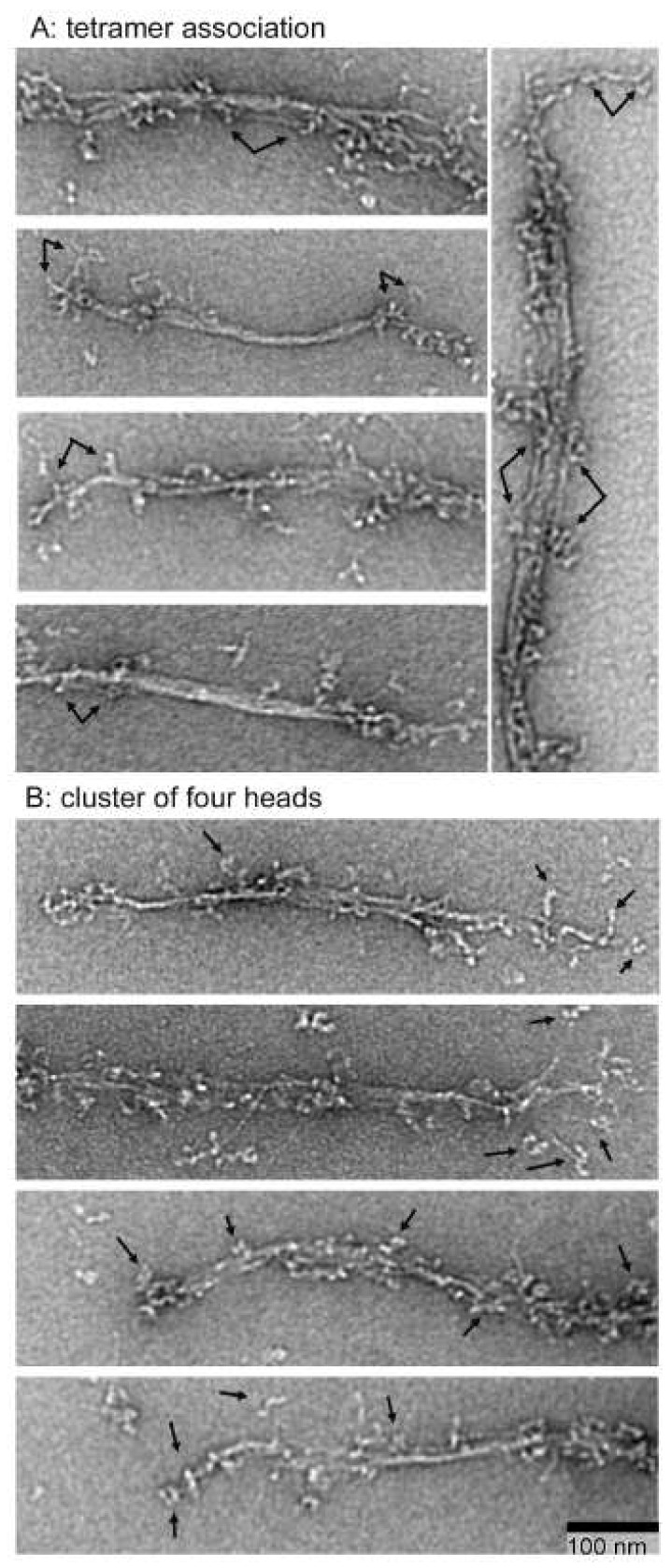
Electron micrographs of growing filaments: (**A**) demonstrates association of folded tetramers, as indicated by arrows with growing filaments; (**B**) shows the clusters of four heads, as indicated by arrows.

The polymerization process proceeds rapidly. Even though the polymerizing filaments were fixed within a few seconds of initiation of polymerization, few free intermediate structures, as described above, were observable, with most structures being growing filaments. Some antiparallel folded tetramers were observed to be associated with growing filaments, with these associations occurring in the middle regions or growing points of the polymerizing filaments (Figure 5A). Multiple tetramers were observed to be simultaneously associated with a growing filament.

**Figure 6:**
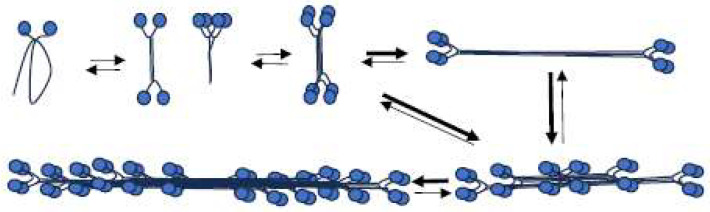
*Dictyostelium* myosin II polymerization pathway. In a solution containing 50 mM NaCl and 2 mM MgCl_2_ (pH 7.0), *Dictyostelium* myosin II monomers adopt a folded structure like their counterparts in muscle and nonmuscle cells. Folded monomers associate to create either folded parallel dimers or antiparallel dimers, ultimately forming folded antiparallel tetramers. These folded tetramers then unfold into extended antiparallel tetramers. The process continues as folded tetramers associate with unfolding tetramers, opening up and growing into filaments. The thick arrows mean fast process in the equilibrium.

Based on these structural features, the inferred polymerization pathway for *Dictyostelium* myosin II can be outlined (Figure 6): under low-ionic-strength conditions, *Dictyostelium* myosin II initially forms folded monomers, which then associate to create either parallel or antiparallel dimers. Two dimers are subsequently associated to form an antiparallel tetramer. Folded structures within these tetramers undergo unfolding, allowing for more folded tetramers to associate and polymerize into filaments. Importantly, this polymerization pathway resembles that of muscle myosin and mammalian nonmuscle myosin IIs [10,11].

The observed intermediate structures suggest that folded tetramers are the principal units of *Dictyostelium* myosin II polymerization, a concept reinforced by observing clusters of four myosin heads on growing myosin filaments (Figure 5B). 

In the polymerization mechanism, polymerization and depolymerization reach equilibrium under specific conditions. The crucial step involves unfolding folded antiparallel tetramers, which bind to growing filaments or other unfolding tetramers. When conditions favor rapid opening, folded tetramers readily associate with growing filaments, leading to filament polymerization until the middle region is fully occupied, forming a thick filament without a bare zone. However, the association of folded tetramers with growing filaments may be unstable. Conversely, if unfolding is disfavored, the bound folded tetramers can easily detach from the growing filaments, preventing the filaments from thickening. This results in the formation of filaments with varied thicknesses and bare zones under different conditions.

### 3.5. Entwining in the Filaments

**Figure 7:**
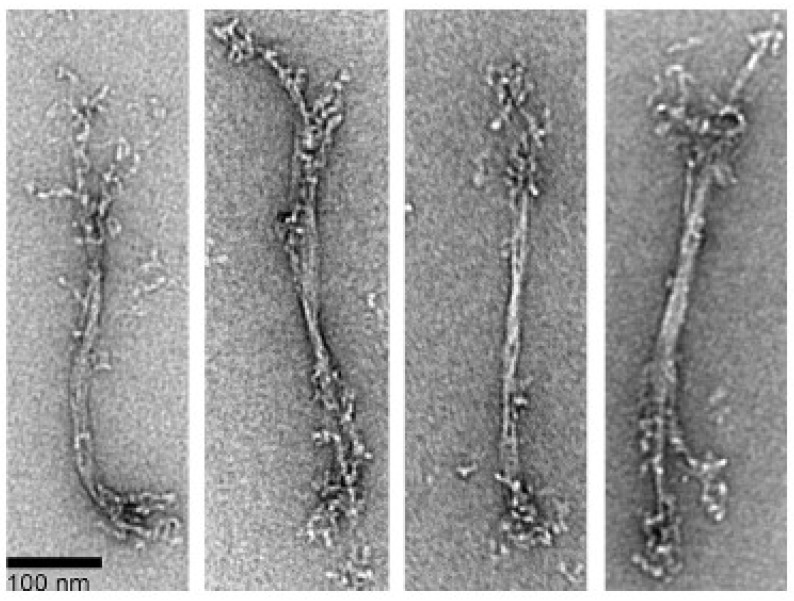
*Dictyostelium* myosin II filaments entwine in bare zones. Myosin was polymerized and immediately fixed with 0.1% glutaraldehyde. Image shows entwining of small filaments in the bare zone of the growing filament.

Myosin polymerization rapidly yields thick filaments, and the quick fixation of polymerizing filaments enables the observation of structural features. Figure 7 illustrates that myosin filaments exhibit twisting in the middle region like mammalian nonmuscle myosin IIs [10], which is believed to result from the entwining of tetramers. Multiple folded tetramers are associated with an extended tetramer or a small filament in various positions and orientations. Once folded tetramers are fully unfolded, they become entwined in the middle region. This twisting within myosin filaments challenges the previous suggestion that filaments form through the lateral association of extended parallel dimers.

### 3.6. Association of Short Filaments to Form Long Filaments

**Figure 8:**
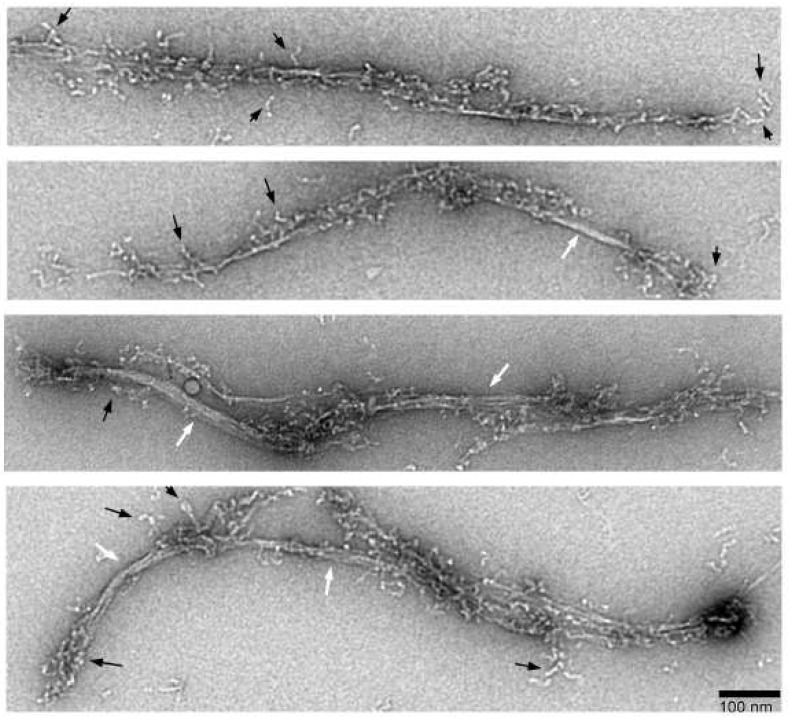
Long filament formation. Myosin was polymerized and immediately fixed. Growing filaments with clean bare zones overlap in the end regions to form long filaments without clean bare zones. Black arrows indicate the cluster of four heads. Some clusters show three visible heads. White arrows indicate bare zones.

In vitro, *Dictyostelium* myosin II forms very long filaments under low-ionic-strength conditions [28], as observed in this study as well (Figure 1 and Figure 2). Figure 8 illustrates that such long filaments are formed through the association of short filaments at the ends, a mechanism similar to that observed with smooth muscle myosin II. As depicted in Figure 8, these filaments are not fully mature and exhibit clean bare zones, as indicated by white arrows. These long structures grow and mature into thick filaments, as demonstrated in Figure 1 and Figure 2, through the association of folded tetramers (presumably at the connection sites too) that unfold and contribute to their growth. The end regions of the filaments overlap and associate, suggesting a mechanism for the rapid polymerization of long filaments. Clusters of four heads can be seen, indicated by the arrow in Figure 8. 

## 4. Discussion

Our study, conducted in 50 mM NaCl and 2 mM MgCl_2_ (pH 7.0), identified folded monomers, folded parallel and antiparallel dimers, and folded and partially opened antiparallel tetramers. We also observed the association of folded antiparallel tetramers with growing myosin filaments and noted entwining in the middle region of growing filaments. Furthermore, we demonstrated through electron microscopy and light scattering assays that ATP significantly reduces the polymerization of *Dictyostelium* myosin II and reduced filament widths. The total ionic strength of amoeboid cytoplasm is estimated to fall within the range of 70–120 mOSM, equivalent to a 35 to 60 mM KCl solution [29]. Consequently, our assembly conditions closely replicate the physiological ionic strength.

Mahajan, R K, and Pardee JD [19] used kinetic and structural analyses to demonstrate that *Dictyostelium* myosin II assembles via a sequential process of slow nucleation and controlled growth, which differs in rate and mechanism from other conventional myosins. This assembly occurs in 50 mM KCl and 10 mM MgCl_2_ (pH 6.5). They observed intermediate structures during polymerization using negative staining electron microscopy, including extended monomers in high-salt conditions, extended parallel dimers, and extended antiparallel tetramers in 50 mM KCl conditions. Based on these structures, they proposed a polymerization mechanism: myosin monomers associate into extended parallel dimers and then into antiparallel tetramers. The lateral addition of dimers to bipolar tetramers completes the assembly of short, blunt-ended, thick filaments. Notably, the thick filaments observed in 10 mM MgCl_2_ were relatively short, approximately 450 nm, considerably shorter than those seen in published images and our images. This suggests that 10 mM MgCl_2_ leads to the over-assembly of myosin II into thick filaments, as shown in *Acanthamoeba* myosin II [30]. In addition, the EM images shown in this study seem to be at odds with the real myosin structures. The measured width of the monomer and dimer tails using the scale bar are about 10 and 20 nm wide, respectively, which are much larger than the actual width of the coiled-coil tail of the myosin II tail (about 2.2 nm). 

Pasternak et al. [15] observed straight parallel dimers in polymerized myosin solutions. Using rotary shadow electron microscopy, they noted the 14 nm staggering of myosin heads in dimers in a no-salt buffer condition (10 mM Tris, 1 mM EDTA, pH 7.5). However, when 60 mM KCl and 2 mM MgCl_2_ were added to the buffer, they observed thick filaments without bare zones and smaller loose filaments. The straight parallel dimers became rare, and some monomers with bent tails appeared to be excluded from the filaments. The folded monomers were not observed in their study. 

Recently, folded monomers of *Dictyostelium* myosin II under low-ionic-strength conditions were observed through negative staining EM and rotary shadowing EM after crosslinking with 0.1% glutaraldehyde [22]. Additionally, these folded monomers were identified in negative staining EM images of heavy chain phosphorylated samples, as depicted in Figure 4b [28], even though not explicitly mentioned by the authors. The absence of observation of folded monomers without crosslinking might be due to the lesser stability of *Dictyostelium* myosin II folded monomers than those of muscle myosin IIs, potentially getting disrupted during sample preparation for shadowing. Considering these findings, it becomes apparent that the polymerization pathway of *Dictyostelium* myosin II in the presence of 50 mM NaCl and 2 mM MgCl_2_ differs from the pathway proposed by Mahajan, R K, and Pardee JD [19,20].

Myosin IIs’ tails can polymerize into thick filaments under low-ionic conditions. *Dictyostelium* myosin II tails form thick filaments, and like the full-length wild type (WT) in situ, they localize into cleavage furrows [31]. Interestingly, when the heads of *Dictyostelium* myosin II are replaced with GFP, resulting in headless myosin, they can still form bipolar filaments [17]. The tail or headless myosin assembly process may differ from the full-length WT. These observations suggest that the monomer conformation may dictate the polymerization pathway.

Regarding the phosphorylation of the regulatory light chain (Ser 13), it activates MgATPase and motor activities [32], aligning *Dictyostelium* myosin II with smooth muscle and mammalian nonmuscle myosin IIs [33]. However, the extent of regulation in *Dictyostelium* myosin II is notably lower than in smooth muscle myosin. Interestingly, while RLC phosphorylation affects both motor activity and assembly in smooth muscle myosin and mammalian nonmuscle myosin IIs in the presence of ATP [27,34,35], our data indicate that RLC phosphorylation does not affect the assembly of *Dictyostelium* myosin II in the presence of ATP.

The phosphorylation of three threonine residues at positions 1823, 1833, and 2029 in the heavy chain inhibits polymerization [14,18,36]. Rotary shadowing EM reveals that *Dictyostelium* myosin II monomers exhibit an extended conformation. Still, the tail bends when the heavy chain is phosphorylated, suggesting that the kink in the tail serves as the polymerization inhibitor. Yet, upon crosslinking *Dictyostelium* myosin II with 0.1% glutaraldehyde, folded monomers were detected in rotary shadowing EM and negative staining EM under low-, but not high-ionic-strength conditions [22]. Notably, this folded monomer phenomenon was not observed with *Acanthamoeba* myosin II and yeast myosin II under the same conditions. These findings collectively suggest that the folded structure of *Dictyostelium* myosin II in low-ionic-strength conditions is genuine, not an artifact induced by glutaraldehyde crosslinking. The observation of the folded structure in the crosslinked sample, but not in the uncrosslinked one, implies that the folded conformation unfolds during sample shadowing. The phosphorylation of the heavy chain appears to promote the folding of *Dictyostelium* myosin II. In the negative staining EM image of the heavy chain phosphorylated sample, careful examination unveils folded monomers, dimers, and a folded tetramer of Figure 4b in [28]. Particularly, a folded tetramer in the upper left corner and a folded monomer (white) in the left middle were very evident. Surprisingly, the authors did not mention these structures, but described them as amorphous aggregates. The current assumption about the inhibitory mechanism contradicts the observation of the folded structure of the *Dictyostelium* myosin II monomer and the structures shown in Figure 4b in [28]. The inhibitory mechanism of polymerization by heavy chain phosphorylation likely involves preventing the unfolding of folded tetramers. 

ATP affects the critical polymerization concentration of *Dictyostelium* myosin II, akin to muscle myosin IIs and nonmuscle myosin IIs in mammals. Of note, our unpublished observations reveal that, under identical conditions, the polymerization of *Acanthamoeba* myosin II and yeast myosin II (myo1) remains unaffected by ATP. These data imply that myosin II, forming folded monomers, experiences alterations in critical polymerization concentration or filament morphology in the presence of ATP. These findings suggest that ATP binding induces a change in the conformation of myosin II heads, likely enhancing the interaction between the heads and the tail region in the folded monomeric state, which, in turn, affects the unfolding process during polymerization. These observations align with EM data indicating that the polymerization of *Dictyostelium*, muscle, and mammalian nonmuscle myosin IIs initiates from folded monomers. 

This study employed glutaraldehyde to capture intermediate structures during Dictyostelium myosin II polymerization. Notably, 0.1% glutaraldehyde effectively fixed *Dictyostelium* myosin II polymers in vitro (Figure 4, Figure 5, Figure 7 and Figure 8). However, it altered the filament morphology (see Figure 1, Figure 2, Figure 7 and Figure 8). Nonfixed myosin filaments exhibited well-extended myosin heads (Figure 1 and Figure 2). Interestingly, *Dictyostelium* myosin II filaments in situ proved susceptible to glutaraldehyde [26]. Cells fixed with 0.025 to 1% glutaraldehyde solution containing 1% formaldehyde, which is conventionally used in thin-section EM, showed no myosin filaments. Yet, myosin filaments were observed with transmission EM when cells were fixed with methanol containing 1% formalin at −15 °C and thin-sectioned. Conversely, synthetic *Dictyostelium* myosin II filaments in vitro could be effectively preserved with glutaraldehyde, as demonstrated in this study.

*Dictyostelium* myosin II rapidly undergoes assembly and disassembly in vivo, taking 35 s to concentrate in pseudopods and 15 s to distribute diffusely into the cytoplasm [37]. Visualizing myosin filaments via TIRF microscopy reveals their structureless nature [19]. In vivo, myosin filaments display relative stability, moving considerable distances at a speed of 0.4 μm/sec. Filament lengths range from 0.4 to 0.95 µm, with a median length of 0.68 µm, consistent with the heterogeneity observed in vitro. Filament lengths measured in situ using TIRF microscopy are notably longer than those obtained with thin-section EM, which showed a length of 0.5 µm [25,26].

Our study delves into the in vitro polymerization process of *Dictyostelium* myosin II. While revealing in vivo polymerization is intriguing, its complexity poses significant challenges, with light microscopy providing limited resolution, and CryoEM’s high resolution suffering from contrast issues and sample damage from irradiation [38]. CryoEM is mostly used for imaging cell membranes rather than the cytoskeleton [39]. Thin-section transmission EM faces challenges in obtaining high-resolution myosin filament structures in fixed cells due to interference from the cytoplasm and other components. Platinum replica electron microscopy (PREM) overcomes these challenges, revealing myosin polymer structures with adequate resolution [40,41]. Since PREM offers insights into the in vivo cell cytoskeleton, its application may validate some structures presented in this study. However, as mentioned above, *Dictyostelium* myosin II filaments are susceptible to glutaraldehyde fixation, which is commonly used in PREM sample preparation. This susceptibility could pose a challenge in obtaining the structure through PREM [26]. 

Myosin II, also known as conventional myosin, features dual heads and an oligomerized tail that forms filaments through association. These myosins are categorized into muscle (smooth, cardiac, and skeletal muscle) and nonmuscle myosin IIs. The latter includes neurons, mammalian nonmuscle cells, invertebrate animals (*C. elegans* and *Drosophila melanogaster*, etc.), lower eukaryotes (*Physarum polycephalum*), and single-cellular organisms (*Acanthaomeba*, *Dictyostelium*, and yeast) [3]. *Acanthamoeba* myosin II was first studied for its polymerization pathway, where extended antiparallel dimers formed and were laterally associated with filaments [9]. Recent research has elucidated the polymerization pathways of muscle and mammalian nonmuscle myosin IIs [10,11]. *Acanthamoeba* myosin II starts with the formation of extended antiparallel dimers. In contrast, muscle and mammalian nonmuscle myosin IIs initiate polymerization from folded monomers, forming antiparallel folded tetramers that unfold and associate into filaments. Investigating the polymerization processes of other myosin II variants may reveal additional mechanisms.

Myosins are evolutionarily conserved proteins found in all eukaryotic cells, ranging from protists to mammals. It has been suggested that all myosins share a common ancestor, consisting of the catalytic head and neck regions [42]. This common ancestry may explain the high sequence and structural similarities within these regions observed in various myosins studied to date, despite the diversity in their tail structures. *Dictyostelium*, as a single-celled organism predating animals, possesses myosin II, which is evolutionarily conserved from single-celled organisms to mammals. Lee et al. have demonstrated that *Dictyostelium*, but not yeast and *Acanthamoeba*, myosin IIs form an interacting-heads motif (IHM) structure like muscle myosin IIs [22]. The IHM is crucial in regulating motor activity to maintain a relaxed myosin II [43,44]. In this study, we demonstrated that *Dictyostelium* myosin II shares filament morphology features with human muscle myosin IIs. Notably, we observed similar intermediate structures (folded monomers, dimers, and tetramers) in *Dictyostelium* myosin II polymerization, contrasting with *Acanthamoeba* and yeast myosin II (unpublished observations). These findings, along with the observed IHM structure [22], indicate a close relationship between *Dictyostelium* myosin II and human myosin IIs, suggesting *Dictyostelium* could be a valuable model for studying disease-related proteins.

## 5. Conclusions

In 2 mM MgCl_2_ and 50 mM NaCl at pH 7.0, *Dictyostelium* myosin II forms bipolar filaments, with or without bare zones. Like muscle myosin IIs, polymerization begins with folded monomers, forming folded dimers (antiparallel and parallel) and tetramers. These tetramers unfold and associate with others to form filaments, which intertwine in the middle region. ATP significantly increases the critical polymerization concentration and decreases filament width. RLC phosphorylation does not affect the polymerization of *Dictyostelium* myosin II, regardless of the presence of ATP.

## Figures and Tables

**Table 1:** Effect of ATP on critical polymerization concentrations (nM) of muscle, mammalian nonmuscle, and *Dictyostelium* myosin IIs.

	No ATP	Plus ATP
Sk myosin	4	6
Ca Myosin	7	13
Sm myosin	2	117
NM 2A	10	80
NM 2B	7	50
NM 2C	3	70
Dcity Myosin II	0.2	9

All critical polymerization concentrations were determined using steady state light scattering. Myosin is polymerized overnight at 4 °C. Non RLC is phosphorylated. The data of muscle myosin II and mammalian nonmuscle myosin II are from our previous studies [11,19]. Sk: skeletal muscle; Ca: cardiac muscle; Sm: smooth muscle; NM 2A: mammalian nonmuscle myosin 2A; NM 2B: mammalian nonmuscle myosin 2B; NM 2C: mammalian nonmuscle myosin 2C.

## Data Availability

Data are contained within the article.
